# Dermatofibromas with Aberrant Expression of CD34 Protein: A Systematic Review and a Reappraisal of Clinicopathological Features and Histogenesis

**DOI:** 10.3390/diagnostics13020185

**Published:** 2023-01-04

**Authors:** Mahmoud Rezk Abdelwahed Hussein, Toka Mahmoud Rezk Abdelwahed Hussein

**Affiliations:** 1 Faculty of Medicine, Assiut University Hospitals, Asyut 2074020, Egypt; 2 Faculty of Medicine, Sohag University, Sohag 1646130, Egypt

**Keywords:** dermatofibroma, CD34, immunohistochemistry

## Abstract

Background: Dermatofibromas (DFs) are benign fibrohistiocytic lesions that usually do not express CD34 protein. This study aimed to analyze the literature concerning the immunohistological and ultrastructural features of DFs. It also related these features to the histogenesis of these lesions. Methods: This study included a PubMed literature search for studies addressing the clinicopathological, ultrastructural, and immunohistochemical features of DFs. It also presented some current cases of CD34-negative DFs and a subset of these lesions with aberrant expression of this protein. Results: Analysis of the PubMed literature revealed that DFs with an aberrant expression of CD34 are rare tumors that commonly affect the extremities of adult females. Separating these tumors from dermatofibrosarcoma protuberans (DFSP, CD34-positive tumors) requires using a large panel of immunostains. Ultrastructurally, DFs are composed of diverse cell types, including cells with histiocytic, myofibroblastic, and fibroblastic features. An analysis of the DFs described by this study revealed that cases with an aberrant expression of CD34 protein had slightly high mean age and male sex predominance when compared to CD34-negative cases. The former commonly affected the extremities. There was no evidence of local recurrence or distant metastasis on follow-up. Conclusions: DFs have the potential to express CD34 protein, defining a rare aberrant phenotype, which was not associated with any differences in the outcome as compared to CD34-negative DFs.

## 1. Background

Dermatofibromas (DFs) are common cutaneous lesions representing approximately 3% of skin biopsies received by dermatopathologists. They are also referred to as benign or superficial fibrous histiocytomas or common fibrous histiocytomas. They are usually seen as asymptomatic firm nodules measuring less than 1.0 cm, mainly on the extremities in women. DFs occur at the ages of 20 years to 40 years old and have female predominance [1]. They are usually centered in the dermis and composed of a variable admixture of spindle-shaped and histiocytic cells that are generally positive for Factor XIIIa, D2-40, and CD68. They are usually negative for CD34 and show variable expression of *α*1-smooth muscle actin, S100, and desmin. Ultrastructurally, DFs consist of cells with fibroblastic, myofibroblastic, and histiocytic features [2,3,4]. The histogenesis of DFs is still controversial. They may represent reactive conditions versus benign neoplasms versus heterogeneous lesions (admixture of reactive and neoplastic components). The vast majority of DFs are benign lesions, with rare cases having a propensity for local recurrence or even distant metastasis that have been reported [1].

There are several histopathological types of DFs. They include common fibrous histiocytoma, myxoid, lichenoid, epithelioid, balloon cell, atrophic, clear cell, cellular, hemosiderotic, aneurysmal, lipidized, atypical, palisading, granular cell, keloidal, lichenoid, and signet ring cell. A single lesion can show histological features of several variants [5,6,7]. Cazzato et al. presented a case of granular cell variant of DF in a 74 years old female who presented with a slowly growing dark-brown papule of the left leg. On histology, DF consisted of fibroblast-like cells and histiocytes with eosinophilic cytoplasm containing granules or microvacuoles. The neoplastic cells were separated by collagen bundles and some inflammatory cell [5]. They were positive for CD68 and negative for Melan A, CD34, CD10, and S100. Ultrastructurally, the granular histiocytes contain lysosomes. The immunohistochemical stains can help separate the benign granular cell tumor from the granular cell variant of DF. In granular cell tumors, the neoplastic cells are positive for S-100, neuron-specific enolase, CD63, and CD68. Alternatively, the neoplastic cells of the granular cell variant of DF are negative for S100 [7].

In 1984, the CD34 antigen (cluster of differentiation 34) was first discovered on hematopoietic stem and progenitor cells. CD34 is a transmembrane phosphoglycoprotein with a molecular weight of approximately 115 kDa. It consists of a heavily sialylated, O-linked glycosylated extracellular domain, a single transmembrane helix, and a cytoplasmic tail [8]. CD34 reacts with several hematopoietic and mesenchymal cells, including endothelial cells, fibrocytes, dermal dendritic interstitial cells, and fibroblastic cells in the connective tissue around blood vessels, nerves, smooth muscle bundles, and hair follicles [9]. CD34 is an antigen associated with fibroblast differentiation in fibroproliferative lesions. It is also usually used to separate CD34-positive tumors, such as DFSP, from CD34-negative lesions, such as DFs.

The connective tissue of most human tissues contains enormous amounts of CD34-positive resident fibrocytes derived from the circulating CD14-positive monocytes. Fibrocytes are not recognized as mesenchymal stromal cells due to their leukocyte-associated cell surface markers, including CD34, CD45, CD80, CD86, and major histocompatibility complex class I and II [10].CD34-positive fibrocytes have the inflammatory features of the histiocytes as well as the tissue remodeling characteristics of the fibroblasts; hence they participate in wound healing and tissue remodeling. They secrete several cytokines and act as antigen-presenting cells and progenitor cells for specific tissues. Ultrastructurally, CD34+ stromal fibrocytes (also known as interstitial dendritic cells) have slender cytoplasmic processes that are closely interwoven with the neighboring fibrocytes, forming CD34+ reticular network [9,10].

Although previous studies have reported the expression patterns of CD34 protein in some DFs [8,9,10,11,12,13,14,15,16,17,18,19], to the best of these authors’ knowledge, minimal research has been performed on DFs with aberrant CD34 protein expression. Additionally, studies that comprehensively assess the literature for the ultrastructural features of DFs and relate them to the histogenesis of these lesions are limited. Therefore, this study was performed to address these issues.

## 2. Methods

**Study design:** This systematic review included a PubMed literature search for the studies describing the clinicopathological, ultrastructural, and immunohistochemical (CD34 protein expression) features of DFs. It also described the expression patterns of CD34 protein in 26 cases of DFs.

**Figure 1:**
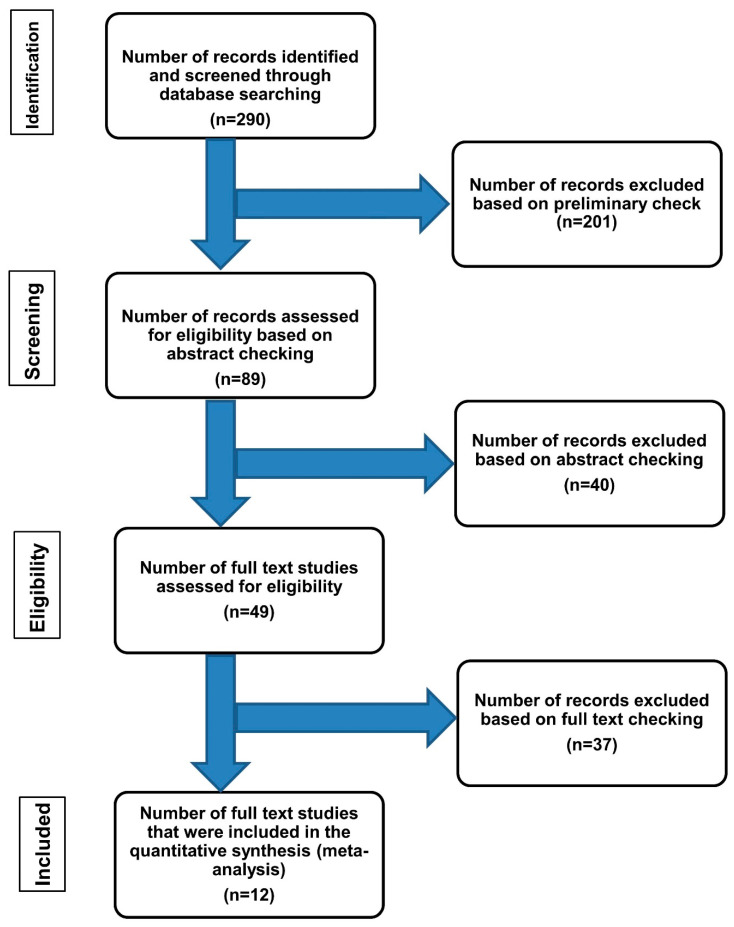
Flow chart of literature search and study selection for cases of dermatofibromas with aberrant expression of CD34.

**A PubMed literature search strategy of the previous studies about DFs, data extraction, and methodological assessment:** To review the PubMed electronic database, the guidelines of PRISMA (Preferred reporting items for systematic reviews and meta-analysis) were followed. The relevant literature for “DFs and CD34” was examined by evaluating the original articles published in peer-reviewed periodicals. The PubMed electronic database was searched using several key terms, including “CD34” and “DF”, or “fibrous histiocytoma”, or “ultrastructure”, or “electron microscopy” to identify the eligible studies. The eligible search results were initially considered based on their titles and abstracts. Their full texts were then examined to confirm their eligibility and included in our study. A summary of the flow trial is presented in Figure 1.

Articles that met all of the following criteria were included in the literature analysis: (i) human studies; (ii) full-length articles published in the English language with keywords “DF or fibrous histiocytoma” being included in the title, or abstract as a final diagnosis”; (iii) cases diagnosed clinically as DFs were initially confirmed by histological examination of the tissue biopsies; and (iv) histological diagnosis was substantiated by immunohistochemical staining (at least CD34 immunostaining was performed) to ensure homogenous evaluation of the findings [11]. Full-length articles, including case reports, case series, and original research articles that did not meet all the inclusion criteria, were excluded from the analysis. Additionally, duplicate publications, conference abstracts, editorial letters, comments, expert opinion papers, review articles, guidelines, consensus, or protocol studies were excluded. The clinicopathological features were extracted from each study. To gain more insights into the histogenesis of DFs, the results of some previous studies about the ultrastructural features of DFs were analyzed. They included 108 cases of conventional, myxoid, myofibroblastic, atrophic, and granular variants of DFs [2,3,4,12,13,14,15].

**Evaluation of the current cases of DFs:** The current DF cases were examined at the Department of Pathology, Assuit University hospitals (courtesy of the corresponding author). Hematoxylin and eosin sections and immunohistochemically stained slides (CD68, CD34, FactorXIIIa, S100, and Ki67 immunostains) were reviewed. The internal positive control consisted of the vascular endothelial cells (dermis). The external positive controls included the case of DFSP known to be positive for CD34 and the stromal cells in the lamina propria (intestine).CD34-positive tumor cells were counted in three randomly chosen square fields (1 mm^2^) following other groups [16]. The percentage of CD34-positive cells was calculated as the number of positive cells per 100 consecutive cells [17,18] and reported as mean values (Mean ± Standard error of the mean, SEM). All slides were coded and evaluated by observers blinded for clinical details and the identity of the patients. The cut-off point applied to consider a case positive versus negative was 5% [19,20,21]. Statistical computations were performed using IBM SPSS 22 (IBM-SPSS Inc., Chicago, IL, USA). The Mann-Whitney test was performed to compare the percentage of CD34-positive cells. It was also used to compare age with CD34 protein expression status. A Chi-square test (**χ^2^**) was performed to compare the gender with CD34 expression status.

## 3. Results

**Figure 2:**
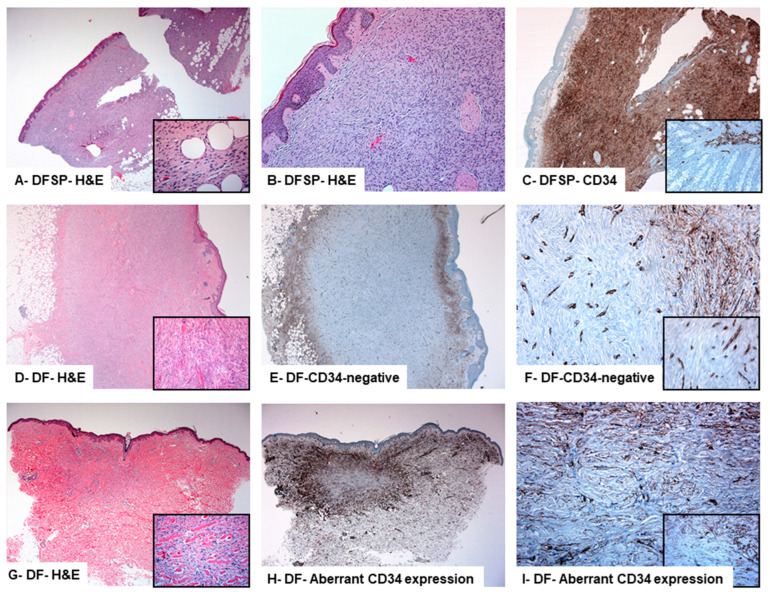
CD34 expression in dermatofibrosarcoma protuberans and dermatofibroma.

**Clinicopathological and ultrastructural features of** DFs **described by the previously published studies:** The PubMed electronic search was performed. It yielded 290 results, of which 201 were excluded from the review. The remaining 89 articles about CD34-positive DFs have undergone “abstract review”. These studies covered a period of 27 years (1992 to 2019). A total of 278 cases (representing 12 studies) with the final DFs/fibrous histiocytoma diagnosis were included. Most cases were negative for CD34 protein expression (227 DFs), whereas only 51 DFs showed aberrant expression of CD34 protein [8,9,10,11,12,13,14,15,16,17,18,19]. Figure 1 represents a summary of the literature search and study selection flow chart. Analysis of the DF cases with aberrant expression of CD34 revealed several observations: (i) the average age was 38 years old [21]; (ii) the lesions were more common in females; (iii) extremities were the most commonly affected sites, refs. [21,22,23,24,25,26,27,28,29,30,31,32]; and (iv) aberrant CD34 protein expression was seen mainly at the periphery rather than the center of these lesions [21]. Some studies suggested that a combination of immunostaining (CD34, D2-40, and factor-XIIIa) is essential to separate these lesions from DFSP [21,24,29]. Other studies indicated that the use of novel immunostains such as insulin-like growth factor-binding protein 7 (IGFBP7), Stromelysin-3 (matrix metalloproteinase family member, MMP-11), CD163 (a hemoglobin scavenger receptor), and HMGA1 and HMGA2 (High-Mobility Group Proteins are architectural transcription factors) [22,25,26,27]. These immunostains were usually positive in most DFs and negative in most DFSP [22,25,26,27]. Tenascin (a matrix glycoprotein) is another new marker that can help differentiate CD34-positive DFs from DFSP. The over-expression of tenascin was observed at the dermal-epidermal junction overlying the lesion in DFs. Alternatively, tenascin protein expression was absent at the dermal-epidermal junction overlying DFSPs [28]. A summary of these studies is shown in Table 1 and Figure 2.

**A-B-C: Histological and immunohistochemical particularities of dermatofibrosarcoma protuberans diagnosed in a 54-year-old female that presented a nodule on the left shoulder.** The tumor is centered in the dermis and subcutis. Grenz zone is involved. It is composed of spindle-shaped cells arranged in storiform to the whorled pattern. The tumor cells are diffusely and strongly positive for CD34 protein expression. A: inset shows the infiltration of the subcutaneous fat by the neoplastic cells. C: inset shows the positive expression of CD34 in the lamina propria of the intestine (positive control). (Original magnifications: A: 100×, inset: 200×; B: 100×; and C: 200×, inset: 400×).

**D-E-F (Case #7): Histological and immunohistochemical particularities of CD34- negative dermatofibroma diagnosed in a 63-year-old female that presented with a hypopigmented, raised lesion on the right leg.** Histologically, the lesion is large and composed of non-encapsulated, dermally centered, densely cellular spindle cell lesion, separated by collagen bundles and arranged in vague bundle patterns. Grenz zone is present. The lesional cells are negative for CD34 protein expression (D: inset). The perilesional tissue shows a rim-like diffuse CD34 positive reaction in the dendritic cells. F: inset showing positive CD34 protein immunolabelling of the endothelial cells versus its complete absence in the lesional spindle-shaped cells (Original magnifications: A: 100×, inset: 200×; B:100×; and C: 200×, inset: 400×).

**G-H-I (Case #3): Histological and immunohistochemical particularities of dermatofibroma with aberrant expression of CD34 protein, diagnosed in a 57-year-old female who presented with a nodule on the skin of the shoulder.** Histologically, there is a small non-encapsulated, dermally centered, fairly cellular spindle cell lesion with cells arranged in a vague fascicular pattern. The spindle cells entrap some dermal collagen bundles (G: inset). Grenz zone is present. The lesional cells are weakly to moderately positive for CD34 protein expression (I: inset). (Original magnifications: A: 100×, inset: 200×; B: 100×; and C: 200×, inset: 400×).

A review of the studies addressing the ultrastructural features of DFs revealed that these lesions are composed of diverse cell types. They include cells with histiocytic, myofibroblastic, and fibroblastic features [2,3,13]. Other features include the presence of pools of mucin (myxoid variant) [12], phagolysosomes and glycogen granules (granular variant) [14], collagen with a mesh-like appearance [4], and phagocytized elastic fibers (atrophic variant) [15]. A summary of these findings is presented in Table 2.

**Figure 3:**
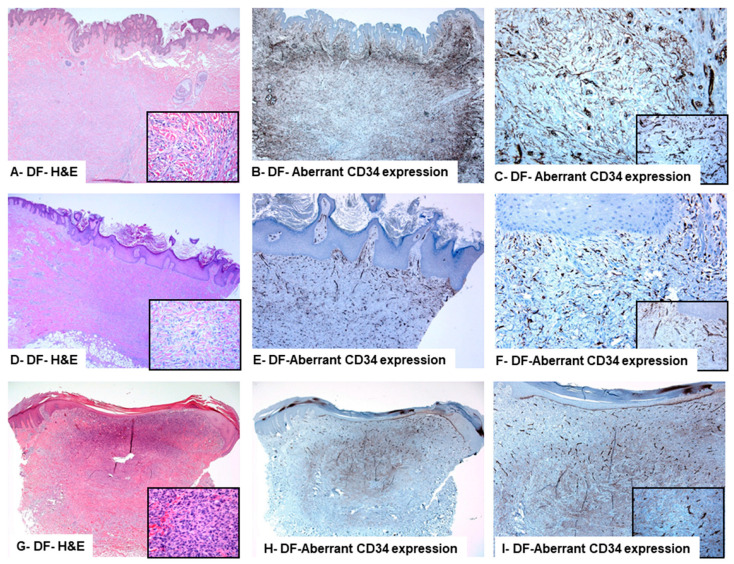
Dermatofibromas with an aberrant CD34 protein expression.

**Clinicopathological features of the DFs described by this study:** All DFs were negative for S100. Alternatively, they were reactive for Factor XIIIa (a marker of dermal dendrocytes), D2-40 (a marker of lymphatic endothelium), and CD68 (a marker of histiocytes). Immunohistochemical analysis of the CD34 protein expression pattern allowed further categorization of these cases into two groups: DFs with aberrant expression of CD34 protein (11 cases) and CD34-negative DFs (15 cases, representing the control group, Figure 2 and Figure 3). Analysis of the clinical findings revealed some variations between the two groups. In the DFs with aberrant expression of CD34, the mean age of the patients (47.5 ± 5.2 years) was high compared to those with CD34-negative DFs (44.5 ± 2.7 years). However, this difference was statistically non-significant. Male sex predominance was noticed in CD34-positive DFs (M: F ratio was 2.6:1) and in CD34-negative DFs (F: M ratio was 4:1). In both groups of DFs: (i) the extremities were the most commonly affected sites, (ii) the average diameter was 0.5 cm, (iii) and follow-up results revealed no local recurrence or distant metastasis two years after diagnosis.

**A-B-C (Case #9): Histological and immunohistochemical particularities of dermatofibroma with aberrant expression of CD34 protein, diagnosed in a 34-year-old male who presented with a lesion on the right shoulder.** Histologically, the overlying epidermis shows verrucous hyperplasia. Within the dermis, the lesion is non-encapsulated, composed of spindle cells, separated by collagen bundles, and arranged in a vague storiform pattern. The cells are separated by collagen bundles (A: inset). Grenz zone is present. The lesional cells are diffusely and moderately positive for CD34 protein expression (C: inset). (Original magnifications: A: 100×, inset: 200×; B: 100×; and C: 200×, inset: 400×).

**D-E-F (Case #6): Histological and immunohistochemical particularities of dermatofibroma diagnosed in a 30-years old male presented with a skin lesion on the left hip**. Histologically, the epidermis shows papillomatosis. The lesion comprises non-encapsulated, dermally centered spindle cells, separated by collagen bundles (D: inset) and arranged in a vague bundle pattern. Grenz zone is present. The lesional cells are focally, weakly to moderately positive for CD34 protein expression (F: inset). (Original magnifications: A: 40×, inset: 100×; B: 100×; and C: 200×, and inset: 400×).

**G-H-I (Case #10): Histological and immunohistochemical particularities of dermatofibroma with aberrant CD34 protein expression diagnosed in a 35-years old male presented with a skin lesion on the left thigh.** Histologically, the epidermis is focally attenuated. A dermally located, non-encapsulated, densely cellular lesion (G: inset) is composed of spindle cells separated by collagen bundles and arranged in vague bundle patterns. Grenz zone is present. The lesional cells are diffusely and moderately positive for CD34 protein expression (I: inset). (Original magnifications: A: 40×, inset: 40×; B: 100×; and C: 100×, and inset: 200×).

Immunohistochemically, no CD34 staining was seen in sections from negative control specimens. CD34 staining was membranous and cytoplasmic. DFs of males expressed significantly higher levels of CD34 protein positively stained cells (40.9 ± 8.3) compared to DFs of females (11 ± 5.6, Chi-square test (χ^2^)**,** *p* = 0.007). There was no association between CD34 protein expression in DFs and the patient’s age (Mann-Whitney U, *p* = 0.646). Low Ki67 labeling indices were seen in both DFs with aberrant CD34 protein expression (labeling index: 0.6%) and CD34-negative DFs (labeling index: 0.4%). DFs with aberrant CD34 expression showed a strong CD34 reactivity at the periphery of the lesions (Figure 2 and Figure 3). Their neoplastic spindle cells at the centers of these lesions showed moderate to strong immunolabelling (Figure 2 and Figure 3). The percentage of CD34-positive cells was significantly higher in CD34-DFs with aberrant CD34 protein expression (54.5 ± 4.0) as compared to CD34-negative tumors (1.7 ± 0.6) (Mann-Whitney U test, *p* < 0.0001). These findings are summarized in Table 3 and Table 4 and Figure 2 and Figure 3.

## 4. Discussion

This study evaluated the literature addressing the clinicopathological [21,22,23,24,25,26,27,28,29,30,31,32] and ultrastructural [2,3,4,12,13,14,15,33] features of DFs It revealed that aberrant CD34 protein expression could occur in DFs. Separating these lesions from DFSPs (CD34-positive tumors) requires using a large panel of immunostains. At the ultrastructural level, DFs are composed of several cell types with diverse differentiation.

The analysis of the previous studies [21,22,23,24,25,26,27,28,29,30,31,32] and the current cases of DFs revealed the lack of CD34 protein expression in some subsets of DFs. This finding supports the ultrastructural studies indicating that the lesional cells in DFs have fibroblastic, myofibroblastic, and histiocytic derivation. All of these cell lineages lack CD34 protein expression [2,3]. This is reminiscent of similar changes in the wound-healing process, which is associated with phenotypic differentiation of CD34-positive dermal interstitial dendritic cells into SMA-positive myofibroblasts under the effects of cytokines [34].

In agreement with previous studies [21,22,23,24,25,26,27,28,29,30,31,32], immunohistological analysis of the DFs presented here revealed a subset with aberrant expression of CD34 protein. Xiong et al. examined the variations between DFs and DFSPs. CD34 reactivity was seen in 5% of DFs. The authors suggested that the lesional cells of DFs are much more reminiscent of mature dermal dendritic cells, whereas those of DFSPs are much more similar to immature dermal dendritic cells [24]. Cohen et al. proposed that this aberrant CD34 reactivity in DFs is due to background or demarcation phenomenon, which occurs when the background contains abundant small, flattened vessels or due to nonspecific staining with CD34 antibodies [35]. In this author’s opinion, the aberrant expression of CD34 in DFs may be due to the presence of cells with diverse phenotypes, including myofibroblasts (CD34-negative cells) and histiocytes (CD34-negative cells) and, to a lesser extent, mature dendritic cells (CD34-positive cells) [36], reflecting multiple lines of derivation or differentiation— an ambiguous histogenesis. This aberrant expression of CD34 may also be due to the persistence of CD34-positive dermal dendritic cells or the transformation of these cells into primitive endothelial cells (CD34-positive cells). The testing of these propositions is open for future investigations.

In all cases of DFs examined by this study, they were reactive for Factor XIIIa (a marker of dermal dendrocytes), D2-40 (a marker of lymphatic endothelium), and CD68 (a marker of histiocytes). These immunophenotyping findings support the ultrastructural presence of several cells with diverse differentiation in DFs. Ultrastructurally, DFs consist of spindled cells intimately related to capillary vessels, with prevailing features of fibroblasts and variable histiocytic, myofibroblastic, endothelial cells, and dermal dendrocytes [33]. These ultrastructural diverse cell types are reflected by variable immunolabelling of the neoplastic cells of DFs for Factor XIIIa (dermal dendritic cells), D2-40 (dermal dendritic cells), *α*1-smooth muscle actin (fibroblasts and myofibroblasts, CD34-negative cells), S100 (dermal dendritic cells), desmin (myofibroblasts), CD34 (endothelial cells and dermal dendritic cells) and CD68 (histiocytes). This ultrastructural diversity of the constituent cells is also reflected by several histological variants of DFs such as cellular, lipidized, epithelioid cell histiocytoma, fibrotic, aneurysmal, granular cell DFs and DFs with monster cells.

**Figure 4:**
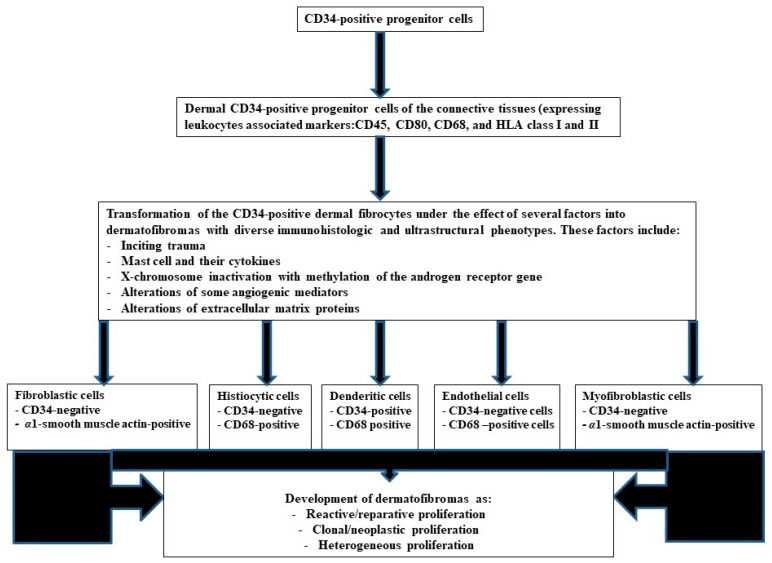
Proposed histogenetic pathway in dermatofibromas.

This author hypothesizes that “the spindle-shaped and histiocytic cells of DFs derive from the modified CD34-positive fibrocytes”. These cells can retain their phenotype (DFs with aberrant CD34 protein expression) or transform to fibroblasts, myofibroblasts (CD34-negative cells), or histiocytes (CD68-positive cells) or undergo a mesenchymal-endothelial transition (CD34-positive cells). This author suggests that some DFs represent a non-clonal (reactive) process that originates following local traumas resulting in fibroblastic stimulation and proliferation. In support, some authorities consider DFs as reactive/reparative lesions that follow inciting trauma to the skin, such as insect bites or superficial punctures. The lack of CD34 protein expression in the DFs possibly reflects the transformation of CD34-positive stromal cells to CD34-negative, *α*1-smooth muscle actin–positive fibroblasts/myofibroblasts [37]. Some DFs may represent a clonal process (neoplasm). In support, molecular changes such as X-chromosome inactivation with the methylation of the androgen receptor gene have been reported in some DFs [38]. Some DFs can undergo malignant transformation [39]. Some sets of DFs represent heterogeneous proliferation comprising reactive/reparative (fibroblastic cells) and neoplastic (histiocytic cells) components [1,2,3,4,12,13,14,15,21,22,23,24,25,26,27,28,29,30,31,32,33]. A summary of this proposed pathogenetic pathway is shown in Figure 4.

## 5. Conclusions

This study indicates that DFs with aberrant CD34 protein expression are rare. Their clinical features and outcomes are similar to CD34-negative DFs. It also demonstrates that using an extended panel of immunostains (such as Factor XIIIa, and D2-40) is required to separate DFs with aberrant CD34 positivity from other CD34-positive lesions, such as DFSPs.

## 6. Recommendations

This study suggests that CD34-positive fibrocytes can transform or differentiate into other cell types (fibroblasts, myofibroblasts, histiocytes, and endothelial cells). Therefore, future cell culture studies with the propagation of dermal fibrocytes, telocytes, and myofibroblasts cell lines to observe their differentiation in vitro culture model may help improve our understanding of the histogenesis of DFs. It is also tempting to examine DFs with aberrant expression of CD34 proteins for the presence of chromosomal translocations and fusion of specific genes, such as the platelet-derived growth factor-beta chain (PDGFB) gene at 22q13 and the collagen type 1 alpha 1 (COL1A1) at 17q22 using fluorescence in situ hybridization (FISH) and multiplex reverse transcriptase-polymerase chain reaction (RT-PCR) [34,40,41]. The authors hope these recommendations open some avenues for future research into the histogenesis of these lesions.

## Figures and Tables

**Table 1:** Data from individual studies of dermatofibroma with aberrant expression of CD34 protein.

Studies	Dermatofibromas with Aberrant CD34 Protein Expression/Total Cases	Contributions of Each Study	References
1	7/30 (23%)	A broad panel of immunostains is needed in DFs with CD34 positivity	[21]
2	4/30 (13%)	A combination of CD34, FXIIIa, and Stromelysin-3 immunostains can separate DFs from DFSPs	[22]
3	4/19 (21%)	The expressions of extracellular matrix proteins play a role in the development of DFs	[23]
4	1/26 (3%)	A combination of CD34 and FXIIIa immune markers can separate DFs from DFSPs	[24]
5	4/23 (17%)	A combination of CD34 and Stromelysin-3 immunostains can help to separate DFs from DFSPs	[25]
6	1/19(5%)	The inclusion of the CD163 marker (hemoglobin scavenger receptor) can help to separate DFs from DFSPs	[26]
7	8/22 (36%)	The use of HMGA1 and HMGA2 (members of the high mobility group protein family genes) immune markers can help to separate DFs from DFSPs	[27]
8	5/20 (25%)	The overexpression of tenascin at the dermal-epidermal junction overlying the lesion in DFs but not in DFSPs helps separate these tumors.	[28]
9	12/30 (40%)	A combination of CD34 and FXIIIa can separate DFs from DFSP	[29]
10	1/13 (7%)	There is no convincing evidence indicating the derivation of DFs from cells with the vascular or hematopoietic origin	[30]
11	2/26 (7%)	Atrophic variants of DFSP and DFs represent distinct entities that can be separated by the use of immunostains such as CD34, Factor XIIIa, and metallothionein	[31]
12	2/20 (10%)	The deep penetrating DFs and DFSP represent distinct entities	[32]

**Table 2:** Data from individual studies about ultrastructural features of dermatofibromas.

Studies	Ultrastructural Findings	Number of Cases of Dermatofibromas	References
1	Spindle cells and dense collagen with the mesh-like appearance	2 cases	[4]
2	Multiple capillary vessels having prominent endothelium and a perivascular ovoid or spindled cells showing intracytoplasmic lipid material and subplasmalemmal densities but lacking cell processes	10 cases	[33]
3	Cells with histiocytic and fibroblastic features	11 cases	[3]
4	Cells with phagocytized elastic fibers	Atrophic variant (a single case)	[15]
5	Fibrocytes amid fibrillary collagen and pools of mucin	Myxoid variant (7 cases)	[12]
6	Cells with abundant endoplasmic reticulum and Golgi complex, several intermediate filaments	Myofibroblastic variant (36 cases)	[13]
7	Cells with pools of phagolysosomes and glycogen granules	Granular variant (5 cases)	[14]
8	Most of the cells are Fibroblast-like and histiocyte-like, showing numerous rough endoplasmic reticulum, free ribosomes, bundles of filaments, macropinocytosis vesicles, and a basement membrane-like material on the outer cell surface. Some cells resembling smooth muscle	9 cases	[2]

**Table 3:** A current group of dermatofibromas with aberrant expression of CD34 protein expression.

No of Case	Age	Sex	Localization	Recurrence or Distant Metastasis			IHC		
					CD34 % of positive cells	Factor XIIIa	D2-40	S100	Ki67
1	57	Male	Upper arm	None	35	+	+	-	0.0%
2	48	Male	Upper back	None	35	+	+	-	1%
3	57	Female	Shoulder	None	60	+	+	-	1%
4	41	Female	Left leg	None	60	+	+	-	1%
5	41	Male	Left forearm	None	60	+	+	-	0.0%
6	30	Male	Left hip	None	75	+	+	-	1%
7	45	Male	Left scapula	None	60	+	+	-	1%
8	53	Female	Foot right	None	35	+	+	-	1%
9	34	Male	Shoulder	None	60	+	+	-	0.0%
10	35	Male	Left thigh	None	60	+	+	-	1%
11	49	Male	Left thigh	None	60	+	+	-	0.0%

IHC: Immunohistochemistry.

**Table 4:** CD34- negative dermatofibromas (the control group in the current study).

Cases	Age	Sex	Site	Recurrence or Distant Metastasis			IHC		
					CD34 % of positive cells	Factor XIIIa	D2-40	S100	Ki 67
1	52	Female	Right deltoid	None	0	+	+	-	0.0%
2	41	Female	Left leg	None	0	+	+	-	0.0%
3	42	Female	Chest wall	None	0	+	+	-	0.0%
4	45	Male	Upper back	None	5	+	+	-	1%
5	81	Female	Upper arm	None	0	+	+	-	0.0%
6	38	Female	Left thigh	None	5	+	+	-	1%
7	63	Female	Right leg	None	5	+	+	-	0.0%
8	52	Male	Left forearm	None	0	+	+	-	1%
9	58	Female	Leg	None	5	+	+	-	0.0%
10	75	Male	Left lower leg	None	0	+	+	-	1%
11	72	Female	Upper back	None	0	+	+	-	0.0%
12	21	Female	Left leg	None	5	+	+	-	1%
13	39	Female	Left leg	None	0	+	+	-	1%
14	10	Female	Right cheek	None	0	+	+	-	0.0%
15	24	Female	Left-arm	None	0	+	+	-	0.0%

IHC: Immunohistochemistry.

## Data Availability

All data and materials are included inside the manuscript.

## Abbreviations

DF	dermatofibroma
H & E	Hematoxylin and eosin
CD34	Cluster of differentiation 34
PRISMA	Preferred reporting items for systematic reviews and meta-analysis
IHC	Immunohistochemistry
IGFBP7	Insulin-like growth factor-binding protein 7
Matrix metalloproteinase family member	MMP-11
HMGA1 and HMGA2	High-Mobility Group Proteins
PDGFB gene	Platelet-derived growth factor-beta chain
COL1A1 gene	Collagen type 1 alpha 1 gene
FISH	Fluorescence in situ hybridization
RT-PCR	Multiplex reverse transcriptase-polymerase chain reaction
